# Monitoring Viable Cells of the Biological Control Agent Lactobacillus plantarum PM411 in Aerial Plant Surfaces by Means of a Strain-Specific Viability Quantitative PCR Method

**DOI:** 10.1128/AEM.00107-18

**Published:** 2018-05-01

**Authors:** Núria Daranas, Anna Bonaterra, Jesús Francés, Jordi Cabrefiga, Emilio Montesinos, Esther Badosa

**Affiliations:** a Institute of Food and Agricultural Technology-CIDSAV-XaRTA, University of Girona, Girona, Spain; University of Georgia

**Keywords:** biological control, viability-qPCR

## Abstract

A viability quantitative PCR (v-qPCR) assay was developed for the unambiguous detection and quantification of Lactobacillus plantarum PM411 viable cells in aerial plant surfaces. A 972-bp region of a PM411 predicted prophage with mosaic architecture enabled the identification of a PM411 strain-specific molecular marker. Three primer sets with different amplicon lengths (92, 188, and 317 bp) and one TaqMan probe were designed. All the qPCR assays showed good linearity over a 4-log range and good efficiencies but differed in sensitivity. The nucleic acid-binding dye PEMAX was used to selectively detect and enumerate viable bacteria by v-qPCR. The primer set amplifying a 188-bp DNA fragment was selected as the most suitable for v-qPCR. The performance of the method was assessed on apple blossoms, pear, strawberry, and kiwifruit leaves in potted plants under controlled environmental conditions, as well as pear and apple blossoms under field conditions, by comparing v-qPCR population estimations to those obtained by qPCR and specific plate counting on de Man-Rogosa-Sharpe (MRS)-rifampin. The population estimation did not differ significantly between methods when conditions were conducive to bacterial survival. However, under stressful conditions, differences between methods were observed due to cell death or viable-but-nonculturable state induction. While qPCR overestimated the population level, plate counting underestimated this value in comparison to v-qPCR. PM411 attained stable population levels of viable cells on the flower environment under high relative humidity. However, the unfavorable conditions on the leaf surface and the relatively dryness in the field caused an important decrease in the viable population.

**IMPORTANCE** The v-qPCR method in combination with plate counting and qPCR is a powerful tool for studies of colonization and survival under field conditions, to improve formulations and delivery strategies of PM411, and to optimize the dose and timing of spray schedules. It is expected that PEMAX v-qPCR could also be developed for monitoring other strains on plant surfaces not only as biological control agents but also beneficial bacteria useful in the sustainable management of crop production.

## INTRODUCTION

The development of new biological control agents (BCA) to prevent crop diseases is receiving considerable attention. The use of BCA is in agreement with the principles and benefits of integrated pest management (IPM), reducing the application of conventional plant protection products. In this context, lactic acid bacteria (LAB), which have been extensively reported as food biopreservatives (1–4), show several features that make them candidate BCA of foliar bacterial plant diseases. Several LAB strains are antagonists of many plant-pathogenic bacteria and fungi (5–7) due to a wide diversity of mechanisms of action, such as the production of organic acids, bacteriocins, and other inhibitory bioactive compounds, or to the competition for nutrients or colonization sites (8). Moreover, some LAB strains have been qualified as generally regarded as safe (GRAS) by the U.S. Food and Drug Administration (FDA) and as having a qualified presumption of safety (QPS) by the European Food Safety Authority (EFSA). Concretely, the Lactobacillus plantarum PM411 strain has been selected in our laboratory as potential BCA due to its broad *in vitro* antagonistic activity against several plant-pathogenic bacteria. This strain synthesizes antimicrobial compounds, such as plantaricins EF and JK, and produces lactic acid, efficiently controlling fire blight disease of pear and apple plants (5, 9).

It is necessary to develop strain-specific quantitative methods for monitoring strain PM411 in the environment in order to study its ecological fitness and to optimize formulations and application strategies in the phyllosphere of plants (10–12). Metagenomic studies in apple trees have revealed the presence of Lactobacillus spp. as components of the phyllosphere (13, 14). Therefore, the monitoring method has to be able to discriminate strain PM411 from other inhabitants of the same species in plants. In addition, since the performance of a BCA requires its colonization and survival on plant surfaces, the development of methods capable of quantifying only viable cells is needed.

Several monitoring methods are commonly used to detect and quantify BCA at the strain level in environmental samples, but in most cases, they are unable to estimate only the viable or culturable population. Culture-based techniques combined with the use of an antibiotic-resistant mutant allow the quantification of a specific strain (11) but may underestimate (ca. 2 or 3 log units) the population size of the BCA under nonconducive conditions (15, 16). This is because some bacteria, including LAB species, such as L. plantarum, may enter in a viable-but-nonculturable state (VBNC) as a survival strategy to cope with environmental stress (17–19). VBNC cells retain some metabolic activity and intact membranes, despite minor changes in their composition (18, 20). In contrast, the population level may be overestimated if monitoring methods based on nucleic acid targets, such as real-time PCR (qPCR), are used, since DNA from viable and dead cells can be indifferently amplified (15, 21, 22). The viability quantitative PCR (v-qPCR) constitutes a method that allows the quantification of only viable cells. The method has been shown to be useful for quantification of viable foodborne pathogenic microorganisms, such as Listeria monocytogenes (23), Escherichia coli O157:H7 (23, 24), Campylobacter spp. (25, 26), and Salmonella spp. (23, 27) in different food matrices (e.g., fresh-cut vegetables, ground beef, chicken, and cooked ham). Viability assessment of LAB has also been studied to enumerate probiotic and starter strains in milk and dairy products (28, 29). Moreover, this methodology has been used to monitor a BCA strain of Pantoea agglomerans in citrus fruit (21).

For the development of a strain-specific v-qPCR assay, first, it is necessary to find a specific molecular marker in the strain that can be identified by comparative genomic analysis or even by fingerprinting techniques, such as randomly amplified polymorphic DNA-PCR (RADP-PCR) or amplified fragment length polymorphisms (AFLP) (30–32). In addition, for selectively detecting and enumerating viable bacteria, different nucleic acid-binding dyes such as propidium monoazide (PMA) or ethidium monoazide (EMA) are used in combination with qPCR (33–35). EMA and PMA can penetrate damaged cellular membranes and intercalate into DNA. Light activation of these DNA-bound molecules results in a covalent linkage preventing PCR amplification of the modified DNA. However, both dyes have some limitations. EMA can cross intact cell membranes of some bacterial species and cause, to some extent, inhibition of PCR amplification of viable cells (33). While PMA is highly selective in penetrating only compromised membranes, it is unable to avoid PCR amplification of nonviable cells with unaltered membranes. A new approach, the PEMAX reagent, has been developed recently to improve the v-qPCR and extend the concept of viability PCR to cells with intact cell membrane structure but also with active metabolism. This new approach consists of using an adequate level of EMA (<10 μM) mixed with PMA (≥20 μM) (36–38). Low levels of EMA can cross intact membranes and are accumulated in cells that lack the metabolic ability to offset its uptake. However, EMA is thrown out from metabolically active cells (36). The combination of EMA with PMA increases the strength of the DNA neutralization when samples contain high levels of dead cells with damaged membranes, but it also avoids amplification of DNA of cells with undamaged membranes and inactive metabolism. After the treatment of the bacterial suspension with PEMAX, DNA from viable cells with intact membrane structure and active metabolism (whether culturable or VBNC) will be free of labeling and then detected by qPCR (37).

To our knowledge, the application of the PEMAX reagent in the v-qPCR approach has been recently used in viability assessment studies for monitoring bacterial pathogens (e.g., *Legionella and Salmonella*) (39, 40) but not for the specific quantification of beneficial bacteria in plant environments.

The aim of the present work was to develop a strain-specific v-qPCR assay using the PEMAX system to detect and quantify viable cells of L. plantarum PM411 in aerial plant surfaces. The method has allowed monitoring of the survival of PM411 after artificial inoculation to plant material under different conditions and plant hosts, in comparison to qPCR and plate counting techniques.

## RESULTS

### Identification of an L. plantarum PM411-specific molecular marker.

**FIG 1:**
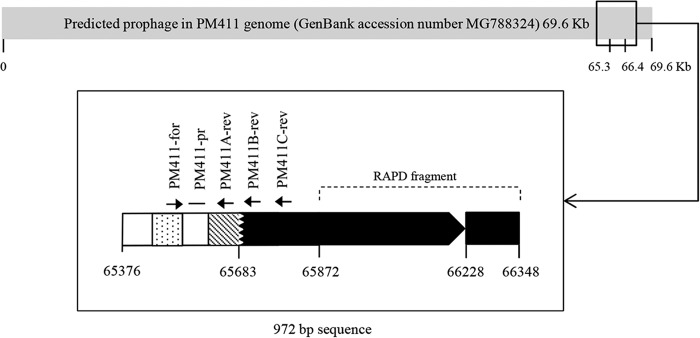
Description of the PM411 sequence (972 bp) in which strain-specific qPCR assays were designed. The sequence was located in the putative prophage (GenBank accession number MG788324) and revealed mosaic architecture. Black boxes show a 665-bp region with 79% identity with 32 L. plantarum strains, encoding a tail fiber and a hypothetical protein. The striped box shows a 90-bp region with ≤85% identity with 7 L. plantarum strains, encoding a tail fiber. The dotted box shows a 162-bp region with 80% identity with L. plantarum HFC8, encoding a hypothetical protein. White boxes show nonhomologous sequences. The primers and TaqMan probe and the region corresponding to RAPD fragment are indicated.

A discriminatory band (520 bp) was found in the PM411 strain by the RAPD-PCR method using the primer XD9 (described in Materials and Methods). Its sequence shared 86% identity with the Lactobacillus phage Sha1 (GenBank accession number HQ141411) and 81% identity with 32 L. plantarum strains available in the NCBI database. Detection for prophage DNA sequences within the PM411 genome using PHAST indicated three regions that were predicted to represent prophages. One of these regions with 69.6 kb (GenBank accession number MG788324) contained the RAPD sequence and was identified as the putative prophage Lactob_PLE3 (GenBank accession number NC_031125) with a score of 150 and with 80 coding DNA sequences (CDS) (see Table S1 in the supplemental material). In particular, the 520-bp sequence (found by RAPD-PCR) was located in the CDS 75 and 76 of the putative phage. Figure 1 shows a 972-bp fragment (from CDS positions 65376 to 66348), which includes the RAPD sequence, with mosaic architecture that contain homologous and nonhomologous sequences to strains available in the NCBI database. Its specificity was confirmed *in silico* since the whole 972-bp fragment was not found in any strain available in the database. The fragment contained homologous sequences, such as 665 bp that shared 79% identity with 32 L. plantarum strains, partially encoding a tail fiber and a hypothetical protein, 90 bp that shared ≤85% identity with 7 L. plantarum strains, partially encoding a tail fiber, and 162 bp that shared 80% identity with L. plantarum HFC8, partially encoding a hypothetical protein. However, the presence of the nonhomologous sequences enabled the identification of a strain-specific molecular marker. The strain specificity of the marker was confirmed by PCR using PM411-for and PM411C-rev primers (Table 1). With this approach, no amplification was obtained for any of the strains listed in Table 2, except for PM314 and PM340, which were confirmed to be clones of PM411 by repetitive element sequence-based PCR (rep-PCR) fingerprinting. L. plantarum PM411, PM314, and PM340 strains showed an identical banding pattern (data not shown). This common banding pattern was clearly different from other L. plantarum strains tested (CM450, FC248, TC92, and WCFS1).

**TABLE 1:** Primers and TaqMan probes used for RAPD-PCR, rep-PCR, and qPCR analysis

Oligonucleotide, primer, or probe by analysis type^*a*^	Sequence (5′–3′)^*b*^	Amplicon length (bp)^*b*^	Reference or source
RAPD-PCR			
P3	CTGCTGGGAC	**—**	72
P4	CCGCAGCGTT	**—**	73
P7	AGCAGCGTGG	**—**	73
M13	GAGGGTGGCGGTTCT	**—**	73
Inva1	GTGAAATTATCGCCACGTTCGGCAA	**—**	74
512Fb	GATGCAGTCGACAATGTGGATGCT	**—**	75
XD9	GAAGTCGTCC	**—**	76
rep-PCR			
ERIC1R	ATGTAAGCTCCTGGGGATTCAC	**—**	70
ERIC2	AAGTAAGTGACTGGGGTGAGCG		
REP-1R	IIIICGICGICATCIGGC	**—**	
REP-2	ICGICTTATCIGGCCTAC		
BOXA1R	CTACGGCAAGGCGACGCTGACG	**—**	
GTG_5_	GTGGTGGTGGTGGTG	**—**	
qPCR			
PM411-for	AGATGCCAGCACTGGATTAAGC		This work
PM411-pr	FAM-TGCACGGCACAACTCAGGCGATT-TAMRA		
PM411A-rev	TTCATAGTAATCCCAGTGGTTTGG	92	
PM411B-rev	CCTTGTCGATACCAAAGTTAGCTATG	188	
PM411C-rev	CGGCGGCACCACCTT	317	

a ERIC, enterobacterial repetitive intergenic consensus sequence; REP, repetitive extragenic palindromic sequence; BOX, BOX sequence; GTG_5_, polytrinucleotide (GTG)_5_ sequence.

b **—**, variable size. Amplicon sizes listed are amplification products obtained by qPCR using PM411-for primer, PM411-pr TaqMan probe, and the corresponding reverse primer (PM411A-rev, PM411B-rev, or PM411C-rev).

**TABLE 2:** Bacterial strains used in this study

Species	Code strain^*a*^
LAB	
Lactobacillus brevis	CECT 4669
Lactobacillus buchneri	CECT 4111^*b*^
Lactobacillus collinoides	CECT 922^*b*^
Lactobacillus dextrinicus	CECT 4791^*b*^
Lactobacillus pentosus	10 strains isolated from plant sources^*c*^
Lactobacillus plantarum	PM411, PM314, PM340, TC54, TC92, FC248, CM450, CM466, RC526, FC534, 35 strains isolated from plant sources^*c*^
	CECT 221, CECT 223, CECT 748^*b*^, CECT 749, CECT 4185, CECT 4308, CECT 4528, CECT 4645, CECT 5785, WCFS1 (syn. of LMG 9211), ATCC 8014
Lactobacillus sakei	CECT 980
Lactococcus lactis	3 strains isolated from plant sources,^*c*^ CECT 539, CECT 984, CECT 4433
Leuconostoc citreum	1 strain isolated from plant sources^*c*^
Leuconostoc fallax	CECT 4701
Leuconostoc mesenteroides	12 strains isolated from plant sources,^*c*^ CECT 219^*b*^
Pediococcus acidilactici	LMG 6411
Pediococcus parvulus	CECT 7350
Pediococcus pentosaceus	LMG 10740
Weissella cibaria	3 strains isolated from plant sources^*c*^
Non-LAB	
Bacillus subtilis	EPS201
Erwinia amylovora	PMV 6076
Escherichia coli	ATCC 5954
Pantoea agglomerans	EPS125
Pantoea vagans	7 EPS strains, C9-1^*d*^
Pseudomonas fluorescens	10 EPS strains, EPS62e
Pseudomonas syringae	7 EPS strains, EPS94
Staphylococcus aureus	ATCC 9144
Xanthomonas axonopodis pv. vesicatoria	2133-2

a CECT, Colección Española de Cultivos Tipo; BCCM/LMG, The Belgian Coordinated Collections of Microorganisms/Laboratory of Microbiology, Ghent University; ATCC, American Type Culture Collection; PMV, Laboratoire de Pathologie Moléculaire et Végétale, INRA/INA-PG, Paris, France; EPS, Escola Politècnica Superior-UdG, Spain; IVIA, Instituto Valenciano de Investigaciones Agrarias, Spain.

b Type strain.

c Trias et al. (4) and Roselló et al. (5).

d Strain provided by F. Rezzonico.

### Strain-specific qPCR designs.

Departing from the PM411 strain-specific marker, TaqMan-based qPCR assays were developed. Three qPCR assays producing different amplicon lengths (92, 188, and 317 bp) were designed in the polymorphic region and checked (A, B, and C) in order to study their suitability in v-qPCR (Fig. 1). The shared forward primer (PM411-for) annealed with the sequence homologous to L. plantarum HFC8, the TaqMan probe (PM411-pr) with the sequence without homology, and the reverse primers (PM411A-rev, PM411B-rev, and PM411C-rev) with the region homologous to several L. plantarum strains (HFC8 nonincluded).

### Specificity, sensitivity, and amplification efficiency of qPCR assays.

At 4 ng of genomic DNA per qPCR (approximately 10^6^ CFU or genomic equivalents), successful amplification of PM411 was achieved, with cycle threshold (*C_T_*) values from 16.5 to 23 by the three strain-specific TaqMan-based qPCR assays developed. No amplification was observed with DNA from pure cultures of the large collection of strains of different species and genera (LAB and non-LAB bacteria) listed in Table 2. Only random fluorescence signals were observed at *C_T_* values higher than 38 in 7 LAB strains and in some plant material washings without PM411 cells. Hence, the three qPCR assays were considered to be specific at the strain level.

Standard curves of the three qPCR assays, which were prepared in flower washings, showed good linearity over a 4-log range (from 1 × 10^3^ to 1 × 10^7^ CFU · ml^−1^, *R*^2^ = 0.99), and the lowest limit of detection was 1 × 10^2^ CFU · ml^−1^. The equations of regression curves for the A, B, and C designs were *C_T_* = −3.3 log CFU · ml^−1^ + 39.7 (for A), *C_T_* = −3.3 log CFU · ml^−1^ + 42.8 (for B), *C_T_* = −3.4 log CFU · ml^−1^ + 47.6 (for C). The corresponding amplification efficiencies (*E*) were 99.9% (A), 98.7% (B), and 98.9% (C). However, the three assays differed in sensitivity. The A design (92 bp) was the most sensitive, followed by the B design (188 bp), with the C design (317 bp) being the least sensitive. The comparison between the three standard curves led to the selection of the A and B assays for further experiments.

### v-qPCR.

**FIG 2:**
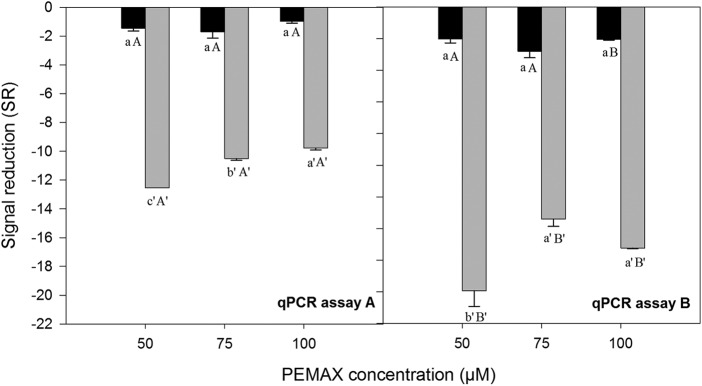
Signal reduction (SR) in viable (black) or dead (gray) cell suspensions with different concentrations of PEMAX reagent (50, 75, and 100 μM). SR is the difference between cycle threshold values (Δ*C_T_*) of non-PEMAX and PEMAX-treated samples. TaqMan-based qPCR assays designated A (92 bp) and B (188 bp) were carried out. The results are shown as means from three independent replicates, and error bars represent standard deviations of the mean. Different capital letters (letters without apostrophe in viable cell suspensions and letters with apostrophe in dead cell suspensions) show significant differences between qPCR assays for each concentration of PEMAX (*P* < 0.05), according to the Tukey test. Different lowercase letters (letters without apostrophe in viable cell suspensions and letters with apostrophe in dead cell suspensions) in the same panel indicate significant differences between concentrations of PEMAX reagent (*P* < 0.05), according to the Tukey test.

The effect of different PEMAX concentrations and qPCR assay (different amplicon length) on the signal reduction (SR) (difference of *C_T_* value [Δ*C_T_*] between non-PEMAX-treated and PEMAX-treated samples) was determined on dead and viable cells (Fig. 2). On dead cells, significant differences of SR between concentrations of PEMAX were observed, and the highest SR in both qPCR assays (A and B) was obtained using 50 μM PEMAX. However, on viable cells, the different PEMAX concentrations did not show significant differences. Based on these results, the PEMAX concentration of 50 μM was chosen for further experiments.

When A and B qPCR assays were compared, on dead cells, a significantly higher SR was obtained by the B assay, with an amplicon length of 188 bp, than by the A assay, with an amplicon length of 92 bp, for all PEMAX concentrations (Fig. 2). On viable cells, only a significant higher SR was obtained with the B than the A assay using 100 μM PEMAX, whereas when using 50 and 75 μM PEMAX, no significant differences between assays were observed. The B assay was finally chosen.

**FIG 3:**
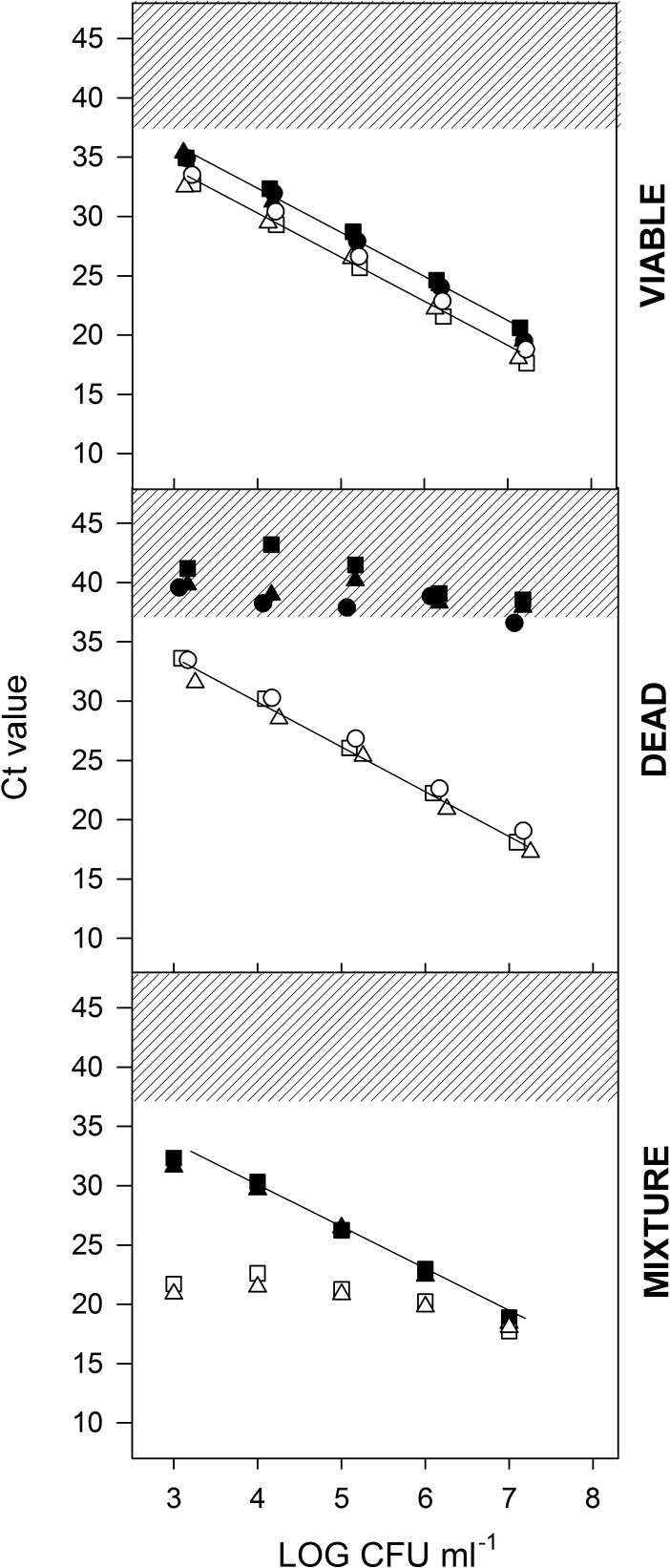
Cycle threshold (*C_T_*) values obtained by TaqMan-based v-qPCR assay (B design) for a range of concentrations from 1 × 10^3^ to 1 × 10^7^ CFU · ml^−1^. The experiment was performed with (i) only viable cells, (ii) only dead cells, and (iii) viable cells in the presence of 1 × 10^6^ CFU · ml^−1^ of dead cells. Cells were treated with PEMAX reagent (black symbols) or not (white symbols) prior to DNA extraction. Three independent experiments represented by circle, triangle, and square symbols were carried out. The striped background represents the detection limit at *C_T_* values of >38.

Standard curves were developed in flower washings to check the v-qPCR method as a specific bacterial detection and quantification tool (Fig. 3). On viable cells, standard curves (each obtained in three independent experiments) were generated using the B assay, with or without PEMAX treatment. The correlation coefficient values (*R*^2^ = 0.99) and the amplification efficiencies (83.5% with PEMAX and 86.2% without PEMAX) were comparable. The standard curves were linear over the range of 1 × 10^3^ to 1 × 10^7^ CFU · ml^−1^, with and without PEMAX treatment. However, in the presence of PEMAX treatment, a shift of 2 cycles was observed regarding the nontreated samples. On dead cells treated with PEMAX, *C_T_* values higher than 38 were obtained over the range from 1 × 10^3^ to 1 × 10^7^ CFU · ml^−1^, meaning that the amplification was inhibited (Fig. 3). In mixtures of viable cells (from 1 × 10^3^ to 1 × 10^7^ CFU · ml^−1^) and a fixed quantity of dead cells (1 × 10^6^ CFU · ml^−1^) treated with PEMAX, the standard curves (each obtained in two independent experiments) achieved a high correlation coefficient (*R*^2^ = 0.98), with an amplification efficiency of 96.9% (Fig. 3). However, the *C_T_* values of this standard curve obtained with the presence of dead cells in the sample were slightly smaller than those from only viable cells, especially when the concentrations of viable cells were low. Without PEMAX treatment, the qPCR assay was unable to differentiate between DNA from viable and dead cells.

### Quantification of viable L. plantarum PM411 in aerial plant surfaces.

**FIG 4:**
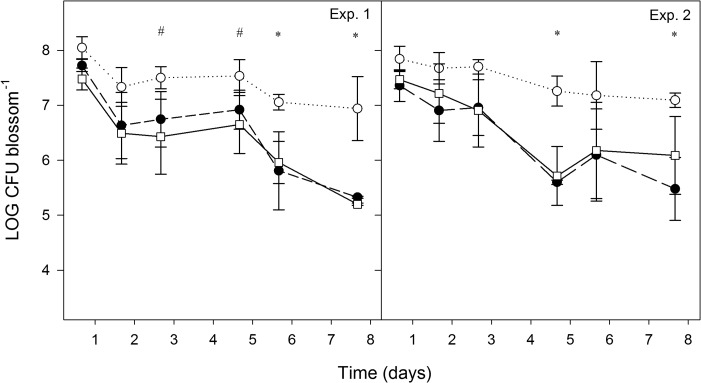
Population dynamics of L. plantarum PM411 estimated by qPCR (total) (○), v-qPCR (viable) (●), and plate counting (culturable) (□) on apple blossoms under controlled-environment conditions (25°C and high rH). Cells were sprayed onto the plant material at 10^8^ CFU · ml^−1^. The experiment was performed two times. Values are the means of three replicates, and error bars represent the standard deviation of the mean. *, significant differences between qPCR and v-qPCR/pc; #, significant differences between qPCR and pc, according to the Tukey test. Exp., experiment.

**FIG 5:**
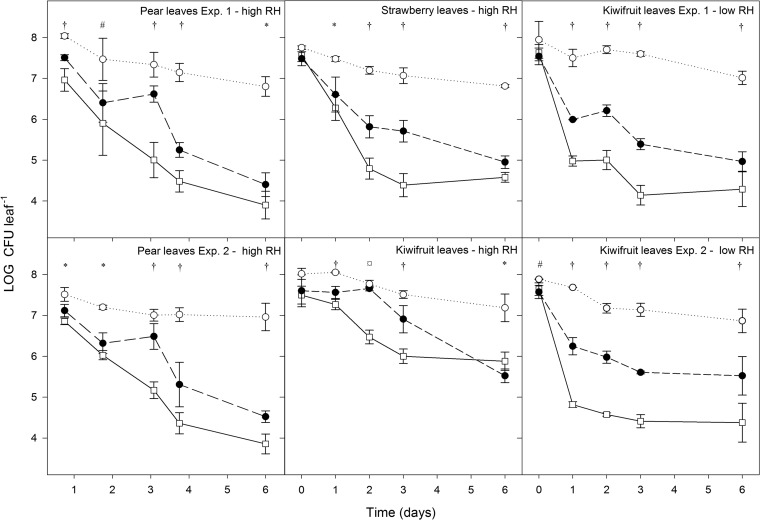
Population dynamics of L. plantarum PM411 estimated by qPCR (total) (○), v-qPCR (viable) (●), and plate counting (culturable) (□) on leaves of pear, strawberry, and kiwifruit plants under controlled-environment conditions (25°C with high or low rH). Cells were sprayed onto the plant material at 10^8^ CFU · ml^−1^. The experiments were performed two times, except for strawberry and kiwifruit plants under high rH. Values are the means of three replicates, and error bars represent the standard deviation of the mean. *, significant differences between qPCR and v-qPCR/pc; #, significant differences between qPCR and pc; †, significant differences between qPCR, v-qPCR, and pc; ¤, significant differences between qPCR/v-qPCR and pc, according to the Tukey test.

PM411 was monitored on inoculated apple blossoms and leaves of pear, strawberry, and kiwifruit plants under controlled environment conditions (25°C, high or low relative humidity [rH]) by qPCR, v-qPCR, and plate counting (pc) (Fig. 4 and 5).

On apple blossoms, in both experiments performed, there were significant differences between qPCR (total cells) and the other two quantification methods, v-qPCR (viable cells) and pc (culturable cells), at some steps throughout the experiments (Fig. 4). After inoculation, the total population size decreased approximately 1 log unit between the 1st and 8th days, whereas the viable and culturable population decreased up to 3 log units. In particular, after a reduction of 1.5 log units during the first 24 h, the viable population remained stable throughout the following 2 days, at around 10^6^ to 10^7^ CFU per blossom, without significant differences compared to the total and cultivable populations. However, after this period, the amounts of viable and culturable cells were significantly lower than total cells. Under these conditions, there was a linear relationship between culturable (pc) and viable (v-qPCR) population levels (*y* = 0.95*x* + 0.12; *R*^2^ = 0.93; *P* < 0.001).

On pear, strawberry, and kiwifruit leaves, significant differences between the three quantification methods (qPCR, v-qPCR, and pc) were observed at some steps throughout the experiments (Fig. 5). Total population level (qPCR) was significantly higher than viable (v-qPCR) and culturable (pc) population levels almost in all sampling days throughout the experiments. Interestingly, significant differences were also observed between the viable and culturable populations, with the quantification of viable cells being significantly higher than the culturable cells, especially after 2 to 3 days under high rH and after only 1 day under low rH. In all the experiments, the total population decreased approximately 1 to 1.5 log units between the 1st and 6th days, whereas culturable and viable cells declined more, from 2 to 4 log units, depending on plant species and rH conditions. While on pear and strawberry leaves at high rH, the population reduction of viable and culturable cells was 3 to 4 log units, on kiwifruit leaves, it was 2 to 2.5 log units. Under low rH, the population decrease of viable and culturable cells on kiwifruit leaves during the 3 days postinoculation (2 to 2.5 log units for viable cells and 3 to 3.5 log units for culturable cells) was higher than under high rH (1 log unit for viable cells and 1.5 log units for culturable cells).

**FIG 6:**
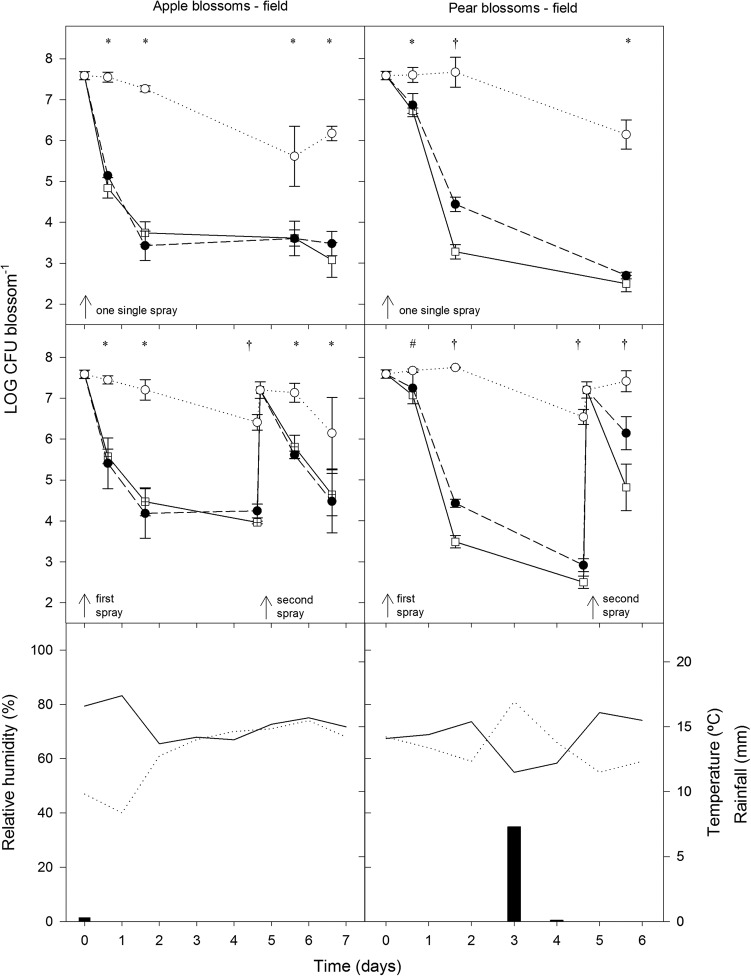
Population dynamics of L. plantarum PM411 estimated by qPCR (total) (○), v-qPCR (viable) (●), and plate counting (culturable) (□) on apple and pear blossoms under field conditions. Cells were sprayed onto the plant material at 10^8^ CFU · ml^−1^. One single spray or two sprays were performed both in pear and apple blossom experiments. Values are the mean of three replicates, and error bars represent the standard deviation of the mean. *, significant differences between qPCR and v-qPCR/pc; #, significant differences between qPCR and pc; †, significant differences between qPCR, v-qPCR and pc; ¤, significant differences between qPCR/v-qPCR and pc, according to the Tukey test. Mean daily temperature (black line), amount of rainfall (black bars), and relative humidity (dotted line) were monitored during the trials.

The population levels of PM411 were also monitored on apple and pear blossoms under field conditions, which were relatively dry (moderate temperatures and low humidity) with one single rainfall event in the pear tree assay (Fig. 6). Total population size differed significantly from viable and culturable population levels at some steps throughout the experiments, both on apple and pear blossoms. Two days following field inoculation (first or single spray), viable cells of PM411 decreased to steady-state values (10^3^ to 10^5^ CFU per blossom) both on apple and pear, without significant differences from culturable cells on apple flowers. However, on pear blossoms, the viable population was significantly higher than the culturable population after 1 to 2 days of PM411 inoculation. When a second spray of PM411 was applied, the three quantification methods (qPCR, v-qPCR, and pc) estimated the same population only immediately after the spray. After 1 day, PM411 population decreased, and significant differences in total population compared to viable and culturable populations were also observed both on pear and apple plants. However, only on pear blossoms was the viable population significantly higher than the culturable population after the second spray. From the different types of plant material and environmental conditions studied, it can be concluded that the populations of viable and culturable cells did not differ significantly under environmental conditions conducive for bacterial survival (on flowers under high rH), but they were different under harsh conditions, especially on leaves under low rH.

## DISCUSSION

Monitoring the persistence and traceability of L. plantarum PM411 in plants is a key task for understanding its behavior in the crop environment and to improve formulations and delivery strategies for biological control of plant diseases. The fate and persistence of target microorganisms in the environment have been traditionally assessed with a variety of culture-dependent and independent methods (6–8, 10, 11). Among the various approaches used, PCR-based methods have been the most popular because they are very sensitive and specific to properly identify the inoculated strains, distinguishing them from the resident population. In the present study, we have developed a viability qPCR assay using PEMAX reagent for the unambiguous detection and quantification of L. plantarum PM411 viable cells in aerial plant surfaces. This method has two main advantages: to be specific at the strain level and to allow the quantification of only viable cells, whether culturable or VBNC.

In order to identify a strain-specific marker, a putative PM411-specific DNA region was identified using the RAPD-PCR technique that showed homology with part of the sequence of Lactobacillus phage Sha1 (41). However, this region was not sufficiently specific to PM411, since it was shared by 32 L. plantarum strains of the NCBI database. The RAPD sequence was located in one of the three prophage regions in the PM411 genome that was predicted to represent the Lactob_PLE3 phage (42). The occurrence of prophage DNA within bacterial genomes is common in LAB, such as Lactobacillus spp. (43–45). This putative prophage in the PM411 genome has mosaic architecture with homologous sequences to L. plantarum strains and also to Lactobacillus, Streptococcus, Bacillus, Enterococcus, and Listeria phages (Table S1), which are alternated with nonhomologous sequences. This polymorphic structure allowed the identification of sequences to design a strain-specific marker. Prophages exhibit a high degree of mosaicism (46) and have been found to contribute to interstrain genetic variability in bacteria (47, 48). Therefore, polymorphic sites within prophage sequences or prophage junction fragments in the genome can be used as indicators of genomic diversity. The presence of homologous phage genes spread in different bacterial strains might reflect phylogeny and suggests horizontal gene transfer between these related species (42). Several studies included the use of phage-related sequences as genomic markers. For example, regions of Lc-Nu and A2 phage sequences were used for strain-specific PCR primer design to identify Lactobacillus rhamnosus strains (49, 50). Moreover, the use of prophage junction fragments as indicators of genomic diversity was already reported in other taxa, such as Salmonella and Listeria (47, 51).

The specificity of the PM411 marker was first confirmed *in silico* and by the absence of amplification signal by PCR in all the L. plantarum strains tested (except for PM314 and PM340, which were deemed to be clones of PM411), other plant-associated bacterial genera, and plant material washings from field samples. Since other strains of L. plantarum may be present in the crop environment (52), this specificity is a key factor in monitoring PM411. Although random amplifications with high *C_T_* values (higher than 38) were observed for some nontarget strains and plant material washings, this phenomenon was previously described in the literature as a background no-template control (15), being irrelevant if remaining outside the range used to generate the standard curve, as occurred in the present study.

The sensitivity and reliability of the three qPCR assays with different amplicon lengths were evaluated mimicking conditions of field sampling by amending plant material washings with different concentrations of PM411, in order to ensure comparable qPCR efficiencies (53, 54). All the qPCR assays fulfill the requirements for satisfactory amplification. Moreover, the values obtained were similar to those reported in other qPCR assays designed to quantify several biological control agents (15, 32, 55).

The use of EMA and PMA coupled with qPCR is an efficient technique to distinguish between viable and dead cells in plant samples (25, 33). These systems have been used to detect foodborne pathogens in lettuce (56) and in fresh-cut vegetables (23), as well as to detect biological control agents in postharvest fruits (21). The new approach based on a double-dye reagent, PEMAX, improves the v-qPCR scope (36–38). In the present study, PEMAX was used to set up a viability qPCR method specific for PM411, and after PEMAX treatment of plant material washings, only DNA from PM411 cells with undamaged membrane and active metabolism was detected by qPCR.

In our work, the effect of PEMAX concentration was optimized to selectively detect viable PM411 cells, avoiding the amplification of heat-killed cells in plant material washings, in accordance with other reports that used PMA dye on different microorganisms (23, 27). According to our study, 50 μM PEMAX in a dead cell suspension in flower washings allowed the inhibition of DNA amplification, while viable cell suspensions were not affected.

In order to choose the best assay for v-qPCR, two designs of different amplicon lengths were compared, taking into account the inactivation of amplification in dead cells, while preserving the performance of qPCR (sensitivity, linearity, and efficiency). Our results showed that the best performance was obtained using the longer amplicon (188 bp). Although the optimal amplicon length for qPCR assays to guarantee method efficiency is less than 100 bp (57), in v-qPCR, longer DNA sequences are necessary (27). As reported, the probability of dye (EMA/PMA) binding in the target region of dead bacteria increases with the length of the DNA fragment (58). Since amplification efficiency and sensitivity of the reaction diminish when amplicon length increases, the reliability of the developed viability qPCR method (using the 188-bp amplicon and PEMAX) was evaluated on viable, dead, and a mixture of viable/dead cells of PM411. The quantification method developed was linear over the range of 1 × 10^3^ to 1 × 10^7^ CFU · ml^−1^, and the obtained standard curves, using *C_T_* values from three independent experiments, showed high correlation coefficient values and amplification efficiencies. Taking into account that the quantification limit was determined in the presence of a high level of dead cells, this sensitivity is similar to those reported in other methods developed to detect and quantify biological control agents (15, 21, 59). The slight increase in *C_T_* values of PEMAX-treated samples compared to nontreated ones was previously reported in studies using PMA as a dye (24, 26). Considering that the PEMAX-qPCR method allowed the quantification of viable cells in the presence of 1 × 10^6^ CFU · ml^−1^ of dead cells with high amplification efficiency, the methodology was suitable to monitor viable PM411 cells in plant samples.

The performance of the method was studied by comparing v-qPCR population estimation to those obtained by qPCR and specific plate counting on de Man-Rogosa-Sharpe (MRS)-rifampin. Since L. plantarum PM411 is a biological control agent of fire blight of apple and pear (5, 9) and is capable of controlling other bacterial plant diseases, such as bacterial canker of kiwifruit and angular leaf spot of strawberry, plant species, like pear, apple, strawberry, and kiwifruit, were used for further experiments to evaluate the method. In addition, different environmental conditions for the BCA, such as blossoms or leaves, under controlled (high and low rH) or field conditions were analyzed. No significant differences were observed between the three methods when the conditions were conducive for bacterial survival. However, under harsh conditions, qPCR quantification overestimated the population level of PM411 (until 4 log units), indicating the presence of nondegraded DNA released from dead cells, and plate counting underestimated the population of the strain (until 2 log units), indicating induction of the VBNC state. Therefore, v-qPCR enabled the most accurate quantification of PM411 viable cells, whether culturable or VBNC, to monitor survival on aerial plant surfaces.

On flowers, under controlled-environment conditions of high rH, PM411 showed a transient drop in population level upon inoculation, probably due to the stressful conditions of the spray. However, after this initial decrease, PM411 attained stable population levels of viable cells for the following 2 days, reaching values from 10^6^ to 10^7^ CFU per blossom. In this period, viable population levels were not significantly different from those estimated by qPCR or plate counting. The usefulness of qPCR or plate counting as monitoring tools of BCA after delivery on plants was also confirmed in the BCA Pseudomonas fluorescens EPS62e that showed efficient colonization of blossoms (15, 60). This result is consistent with the fact that the flower environment is favorable for bacterial survival and colonization because of a high level of nutrients. Sugars, such as glucose and fructose, and amino acids, such as proline, asparagine, glutamic acid, and glutamine, are predominant in apple and pear flowers (61). It is expected that L. plantarum can reach stable populations in the flower environment, since it has the capacity to use a broad range of carbohydrates and amino acids (62). After 5 days on the flower surface, the population of PM411 clearly decreased, coinciding with the end of the life span of flowers. This survival reduction may be attributed to the nonconducive conditions as a result of the senescence of the tissues (13). At this period, population was overestimated by qPCR, indicating the presence of nondegraded DNA being released from dead cells. Studies conducted by other authors monitoring bacteria on plant surfaces confirmed the differences in population estimation between qPCR and plate counting (15, 21). Interestingly, all viable cells were culturable probably because conditions were not enough stressful to induce a VBNC state.

On the leaf surface, the PM411 population decreased more than on flowers after inoculation. The leaf environment is poor in carbon-containing nutrients and more exposed to fluctuations in temperature, UV radiation, and especially water availability (relative humidity and leaf wetness) (63, 64). Under these stress conditions, the induction of a VBNC state and cell death may explain significant differences between v-qPCR, qPCR, and plate counting. However, these differences were observed in leaf experiments both under high and low rH, probably meaning that the lack of nutrients is one of the most important limiting factors for PM411 survival. As reported in other bacteria, including LAB, stressful conditions (e.g., desiccation and starvation) can promote cells to enter in a VBNC state (17, 20). Consequently, VBNC cells were not quantified by plate counting, and the viable PM411 population was underestimated, as reported in other BCA monitoring studies under different stress conditions (15, 16, 65). As VBNC cells are still metabolically active and preserve membrane integrity, they should be considered the effective population of BCA since they can become culturable again when better conditions arrive (20). On flowers under field conditions, the viable population of PM411 dropped to around 10^3^ to 10^5^ CFU per blossom immediately after the spray (both in the single- or two-spray experiments). Although the nutritional conditions in flowers are expected to be optimal, the harsh environment, such as periods of low relative humidity (<70%) combined with UV radiation exposition, probably caused this decrease in population. Interestingly, in a pear orchard, differences between v-qPCR and plate counting were observed, presumptively due to the induction of the VBNC state as an adaptive stress response of cells against suboptimal environmental conditions. It was reported in previous studies that L. plantarum PM411 increased transcript levels of stress-related genes under desiccation (66). However, in an apple orchard, similar populations of viable and culturable PM411 cells were observed. Therefore, differences in morphology and physicochemical environment between pear and apple flowers, as well as weather conditions registered in the field, may explain how a discrepancy between viable and culturable populations was only observed in apple flowers.

In our study, the unfavorable environmental conditions on the leaf surface and the relatively dry field conditions during the experiments seem to induce a VBNC state in PM411 cells.

Finally, we have developed a method for the specific detection and quantification of viable PM411 that has been evaluated and validated. The method is expected to be a reliable monitoring tool to estimate the viable population of the strain in aerial plant surfaces, and it will allow further studies of colonization and survival under field conditions, as well as improvements in formulations and delivery strategies. Data obtained from v-qPCR monitoring may indicate when PM411 should be released in the field to achieve the population required for biocontrol, since the decrease in the viable population can compromise the BCA efficacy. It is expected that PEMAX v-qPCR could also be developed for monitoring other bacterial strains on plant surfaces, not only biological control agents, but also other beneficial bacteria (e.g., biofertilizers and biostimulants) useful in the sustainable management of crop production.

## MATERIALS AND METHODS

### Bacterial strains, growth conditions, and DNA extraction.

The bacterial strains used in this study are listed in Table 2. LAB strains were grown on de Man-Rogosa-Sharpe (MRS) agar (Oxoid, Basingstoke, Hampshire, UK) at 23°C for 48 h. Non-LAB strains were grown on Luria-Bertani (LB) agar at 25°C for 24 h. Escherichia coli DH5α calcium competent cells were used for cloning procedures and were grown in LB medium at 37°C. A spontaneous mutant of wild-type L. plantarum PM411 resistant to rifampin, obtained as previously described (5), was used in this study. All strains were stored in 20% glycerol at −80°C. DNA was extracted according to the method described by Llop et al. (67) from pure bacterial suspensions (Table 2).

### Strategy to identify a strain-specific molecular marker for L. plantarum PM411.

All primers, PCR mixtures, and PCR conditions used in this study are described in Tables 1 and 3, and amplified fragments were analyzed using standard methods.

**TABLE 3:** Amplification mixture and PCR conditions

PCR approach	Amplification mixture^*a*^	PCR conditions^*b*^
RAPD-PCR	1× PCR buffer, 1.5 mM MgCl_2_, 0.2 mM dNTPs, 200 nM each primer, 3.75 U *Taq*, and 100 ng DNA (reaction vol, 50 μl)	For M13: 94°C for 3 min; 35 cycles of 94°C for 1 min, 40°C for 20 s, ramp to 72°C at 0.6°C/s for 20 min; elongation at 72°C for 2 min
		For P3 and P4: 94°C for 3 min; 30 cycles of 94°C for 1 min, 36°C for 2 min, 72°C for 2 min; and elongation at 72°C for 2 min
		For P7, Inva1, 512Fb, and XD9: 94°C for 4 min; 45 cycles of 94°C for 1 min, 35°C for 1 min, 72°C for 1 min; and elongation at 72°C for 5 min
pGEM-T insert amplification	1× PCR buffer, 1.5 mM MgCl_2_, 0.2 mM dNTPs, 200 nM primers T7 and Sp6, 1.875 U *Taq*, and 2 μl recombinant vector (reaction vol, 25 μl)	98°C for 2 min; 35 cycles of 98°C for 10 s, 45°C for 30 s, 72°C for 30 s; and elongation at 72°C for 12 min
PCR	1× PCR buffer, 3 mM MgCl_2_, 0.2 mM dNTPs, 200 nM PM411-for and PM411C-rev, 1.75 U *Taq*, and 25 ng DNA (reaction vol, 25 μl)	95°C for 5 min; 30 cycles of 95°C for 45 s, 60°C for 40 s, for 72°C 40 s; and elongation at 72°C for 10 min
rep-PCR	1× PCR buffer, 1.5 mM MgCl_2_, 0.2 mM dNTPs, 1.6 mg/ml BSA, 10% DMSO, 500 nM each forward and reverse primer for rep-PCR and ERIC-PCR or of the single primer for BOX-PCR and GTG_5_-PCR, 2.5 U *Taq*, and 50 ng DNA (reaction vol, 25 μl)	95°C for 7 min; 30 cycles of 94°C for 1 min, 52°C for 1 min for ERIC- and BOX-PCR or 42°C 1 min for REP- and GTG_5_-PCR, and 65°C for 8 min; and elongation at 65°C for 16 min
qPCR	1× TaqMan universal PCR master mix, 500 nM each forward and reverse primer, 200 nM GP probe or 250 nM RP1 or RP2 probe, and 20 ng DNA or 4 μl DNA sample (reaction vol, 20 μl)	50°C for 2 min; 95°C for 10 min; 50 cycles of 95°C for 15 s and 60°C for 1 min

a dNTPs, dinucleoside triphosphates; BSA, bovine serum albumin; DMSO, dimethyl sulfoxide; *Taq*, *Taq* DNA polymerase (Invitrogen). TaqMan Universal PCR master mix is manufactured by Invitrogen.

b PCR was carried out in a GeneAmp PCR system 9700 (Applied Biosystems) and qPCR in a 7500 real-time PCR system (Applied Biosystems).

RAPD-PCR analysis was carried out to identify a PM411 strain-specific molecular marker. Seven primers were tested in strain PM411 and other selected L. plantarum strains (ATCC 8014, CECT 223, CECT 4528, CECT 5785, TC54, TC92, and WCFS1) by PCR. A potential PM411 strain-specific RAPD band was excised from the agarose gel, purified with a QIAquick gel extraction kit (Qiagen, Hilden, Germany), cloned into the pGEM-T Easy vector (Promega, Madison, WI, USA), and transformed into E. coli DH5α calcium competent cells. Cells were selected by antibiotic resistance in LB agar supplemented with 100 μg · ml^−1^ ampicillin (Sigma, MO, USA), and a PCR analysis with the universal primers T7 and Sp6 was done to confirm the insertion. The RAPD-PCR fragment was sequenced (Macrogen, Seoul, South Korea) and analyzed using the FinchTV 1.4.0 software (Geospiza, Seattle, WA, USA) and Multalin software (68). The specificity was ensured *in silico* using the BLAST program at the NCBI database (http://www.ncbi.nlm.nih.gov/BLAST).

As the fragment identified by RAPD-PCR showed similarity with a prophage, raw data of sequenced L. plantarum PM411 genome were used for prophage region search and annotation using a phage finding tool (PHAge Search Tool [PHAST] [69]). The corresponding putative phage sequence was deposited in the GenBank database (accession number MG788324). The RAPD sequence was located in the putative prophage of L. plantarum PM411, and a 972-bp region (region from positions 65376 to 66348 of accession number MG788324) was checked *in silico* for specificity. This region was used in order to design a primer pair (PM411-for and PM411C-rev) using Primer-BLAST (Table 1). The primers were designed in the PM411-specific region. The specificity of the primers was tested using strains described in Table 2. The rep-PCR amplifications of PM411 and other L. plantarum strains listed in Table 2 (CM450, FC248, PM314, PM340, TC92, and WCFS1) were carried out for clone detection with the repetitive sequence-based oligonucleotides corresponding to ERIC, REP, BOXA1R, and GTG_5_ (Table 1) (70).

### Strain-specific qPCR designs and specificity, sensitivity, and amplification efficiency evaluation.

Three TaqMan-based qPCR assays were designed (Table 1) within the strain-specific marker (region from positions 65376 to 66348 of accession number MG788324) to obtain three amplicons with different lengths. All of them shared the same forward primer (PM411-for) and probe (PM411-pr), but they had three different reverse primers (PM411A-ref, PM411B-rev, and PM411C-rev). Probes were labeled with the 6-carboxyfluorescein (FAM) reporter dye at the 5′ end and with the 6-carboxytetramethylrhodamine (TAMRA) quencher dye at the 3′ end. Primers and TaqMan probes were designed using the Primer Express 3.0 software (Applied Biosystems, Foster City, CA, USA).

The specificity of the qPCR designs was tested after optimization of the concentrations of the primers and probe using bacteria listed in Table 2. A no-template control (NTC), using water instead of genomic DNA, and positive control with PM411 DNA were included in all PCR runs. All reactions were performed in triplicate and were carried out in a 7500 real-time PCR system (Applied Biosystems, Foster City, CA, USA).

Standard curves were developed to check the sensitivity and efficiency of the qPCR assays by mixing several concentrations of PM411 cells with plant material washings. To obtain plant material washings, open blossoms of apple (cv. Golden Smoothee) and pear (cv. Comice) and leaves from potted plants of pear (cv. Conference), strawberry (cv. Darselect), and kiwifruit (cv. Hayward) were used. Two blossoms or three leaves were infused with 30 ml of 50 mM sterile phosphate buffer (PBS; pH 7.0) and 0.1% peptone in a 100-ml bottle and mixed in an orbital shaker (KS501 digital; IKA Labortechnik, Staufen, Germany) at 130 rpm for 30 min on ice (15, 60). Cell suspensions of PM411 were prepared in sterile distilled water at high concentration (10^9^ CFU · ml^−1^) and diluted to appropriate concentrations with plant material washings. The cell concentration of the first serial decimal dilution was checked by a measure of the optical density at 600 nm (OD_600_), considering that 0.25 corresponds to 10^8^ CFU · ml^−1^, and this was confirmed by colony counts. The tested concentrations covered a 5-log range, from 1 × 10^3^ to 1 × 10^8^ CFU · ml^−1^. An aliquot of plant material washings without PM411 cells was kept as a no-template control (NTC) sample.

DNA was isolated according to the method described by Schmidt et al. (71), with some modifications. Briefly, 1 ml of sample was centrifuged at 13,200 × *g* for 10 min, and the pellet was resuspended in 400 μl of TES buffer (50 mM Tris-HCl, 1 mM EDTA, 6.7% glucose). Cells were incubated with 100 μl of lysozyme at 20 mg · ml^−1^ (Sigma) and 6 μl of mutanolysin at 5,000 U · ml^−1^ (Sigma) for 1 h at 37°C with shaking (ThermoMixer F1.5; Eppendorf, Hamburg, Germany). After adding 15 μl of proteinase K at 20 mg · ml^−1^ (Qiagen) and 40 μl of 20% sodium dodecyl sulfate (SDS), samples were incubated at 60°C for 1 h. Then, mechanical disruption was performed transferring the sample into a 2-ml safe-seal microcentrifuge tube with 70 mg of acid-washed glass beads (Sigma) and using a TissueLyzer II instrument (Qiagen) at a frequency of 30 s^−1^ for 10 min. Glass beads and cell debris were removed by centrifugation at 12,000 × *g* for 10 min, and the supernatant was purified adding 1 volume of phenol-chloroform-isoamyl alcohol (25:24:1) (Sigma) and mixed by vortexing for 5 s. Phases were separated by centrifugation at 16,000 × *g* for 5 min. The aqueous phase was mixed with 2 volumes of ice-cold ethanol and 0.1 volume of 4 M sodium acetate, and DNA was precipitated overnight at −20°C. DNA was collected by centrifugation at 16,000 × *g* for 30 min, and the pellet was washed in ice-cold 70% ethanol, dried, resuspended in 50 μl of water, and stored at −20°C until analyzed. The amount and purity of DNA samples were determined spectrophotometrically (NanoDrop ND-1000 spectrophotometer; Thermo Fisher Scientific, USA).

qPCR was performed, and two no-template controls (NTC) were included in all PCR runs: (i) one using water instead of genomic DNA, and (ii) one using DNA isolated from plant material washings without PM411 cells. All reactions were performed in triplicate as described above. *C_T_* values were plotted against the logarithm of the initial number of CFU · ml^−1^, and standard curves were generated by a linear regression of the plotted points. Slopes were used to determine the amplification efficiency of each design using the equation *E* (%) = (10^−1/slope^ − 1) × 100.

### v-qPCR. (i) PEMAX concentration optimization.

PEMAX reagent (GenIUL, Terrassa, Spain) was diluted in 500 μl of sterile bidistilled water to obtain a stock solution of 2,000 μM that was stored at −20°C in the dark until needed. To determine the optimal concentration of PEMAX (50, 75, and 100 μM), an appropriate volume of PEMAX stock solution (25, 37.5, or 50 μl) was added into 1 ml of viable or dead PM411 cell suspension, both adjusted to 1 × 10^6^ CFU · ml^−1^ in apple flower washings. Dead cells were obtained by heating a cell suspension at 100°C for 15 min (10, 26) (ThermoMixer F1.5; Eppendorf). The loss of cell viability was checked by plating on MRS agar, followed by incubation for 48 h at 30°C. Then, samples were thoroughly mixed and incubated for 30 min in the dark at room temperature in an orbital shaker KS501 digital (IKA Labortechnik) at 130 rpm. Immediately, samples were photoactivated for 15 min with the PhAST Blue photoactivation system (GenIUL, Barcelona, Spain) set to 100% and transferred into DNA low-binding 1.5-ml tubes (Sarstedt, Nümbrecht, Germany). PEMAX-treated cells (viable and dead) were collected by centrifugation at 13,200 × *g* for 10 min and washed with 50 mM sterile PBS (pH 7.0) under the same centrifugation conditions. Samples of viable and dead cells, adjusted to 1 × 10^6^ CFU · ml^−1^ in apple flower washings and without being treated with PEMAX were also used. DNA extraction of PEMAX-treated and non-PEMAX-treated samples was performed as described in the previous paragraph. Each experimental condition was assayed in two independent experiments.

### (ii) Amplicon length effect.

To study the influence of amplicon length in the effectiveness of the PEMAX treatment to suppress PCR amplification of dead cells, two independent qPCR assays (A and B) were performed. One unique forward primer (PM411-for) and probe (PM411-pr) and two reverse primers (PM411A-rev and PM411B-rev) were used to obtain two amplicons with different lengths (Table 1). qPCR was performed as described previously, and a no-template control (NTC) was included in each PCR run. All reactions were performed in triplicate.

The effect of PEMAX at different concentrations on DNA amplification suppression by qPCR assays A and B was tested in viable and dead cells and expressed as “signal reduction.” Signal reduction was calculated by subtracting *C_T_* values between non-PEMAX-treated and PEMAX-treated samples. Three independent experiments were performed.

### (iii) Standard curve.

To check the v-qPCR method as a specific bacterial detection and quantification tool, the sensitivity and amplification efficiency of the v-qPCR B design were evaluated by developing standard curves. Cell suspensions were prepared using viable, dead, or mixtures of PM411 cells in apple flower washings. Samples were prepared from covering a 5-log range (from 1 × 10^3^ to 1 × 10^8^ CFU · ml^−1^) of viable or dead cells, obtained as described, and mixing the same concentration range of viable PM411 cells with a constant number of dead cells (1 × 10^6^ CFU · ml^−1^). From each sample, 1 ml was treated with PEMAX at 50 μM according to the procedure described previously. An aliquot of each sample without being treated with PEMAX was also used. DNA extraction was performed in PEMAX-treated and non-PEMAX-treated samples as described above. qPCR was performed using the B design (PM411-for, PM411-pr, and PM411B-rev), obtaining an amplicon size of 188 bp. qPCR was performed as described previously, including the two negative controls (NTC) mentioned above. All reactions were performed in triplicate. Standard curves were generated as described above. Three independent experiments were carried out.

### Quantification of L. plantarum PM411 on plant material.

Greenhouse experiments were performed in different plant materials, such as apple blossoms and pear, strawberry, and kiwifruit leaves. Two field trials on apple and pear blooming trees at the Mas Badia Agricultural Experimental Station (Girona, Spain) were also included.

Greenhouse experiments on leaves were conducted in potted plants (10-cm-diameter plastic pots) of pear (cv. Conference), strawberry (cv. Darselect), and kiwifruit (cv. Hayward). Plants were used when they were 30 to 40 cm in length, with 6 young leaves per shoot in pear plants, 10 to 15 young leaves in kiwifruit plants, and 5 to 8 leaves per crown in strawberry plants. Open blossoms of the Golden Smoothee apple cultivar were obtained from a commercial orchard near Girona (Spain). Flowery branches were collected and transported to the greenhouse under refrigeration, and the lower end of the branches was kept submerged in a 5% sucrose solution. To inoculate PM411, the plant material was sprayed to runoff (10 ml per pear plant, 6 ml per strawberry plant, 20 ml per kiwifruit plant, and 3 ml per open blossom) with the bacterial suspension at 10^8^ CFU · ml^−1^. All plant material was kept at 25°C, with a 16-h fluorescent light/8-h dark photoperiod. Treated flowery branches and potted plants were covered with plastic bags to reach high-rH conditions. A group of kiwifruit plants was maintained at low rH (70%) in controlled-environment chambers (SGC097.PFX.F; Fitotron, Sanyo Gallenkamp plc, UK). The experimental design consisted of three replicates of four pear potted plants, three strawberry and kiwifruit plants, or five flowery branches containing 15 blossoms in total. Experiments were carried out twice, except for strawberry and kiwifruit plants at high rH, which were performed once. Sampling for monitoring the PM411 population was performed immediately or at 12 h after inoculation and over six (plant assays) or eight (flower assays) days. Two blossoms, three leaflets of strawberry plants, and three leaves of pear or kiwifruit plants were taken from each replicate and sampling date.

Field experiments were conducted in plots of cv. Golden Smoothie apple and cv. Comice pear trees during the blooming period. Three replicates of 7 trees per replicate were used. In each tree, two branches containing blossoms were tagged. Two strategies were assayed in independent experiments: one doing a single application of PM411 to trees (day 0), and a second strategy with two applications (days 0 and 5). Open blossoms from tagged branches were spray inoculated until near runoff with the bacterial suspension at 10^8^ CFU · ml^−1^ using a handheld 5-ml sprayer (3 ml per blossom). Temperature, rH, and rainfall were measured with a weather station located in the experimental field (Mas Badia, La Tallada d'Empordà, Girona, Spain). Sample collection for monitoring PM411 levels was performed at 0, 1, 2, 5, 6, and 7 days. Samples of two blossoms (4 to 6 flowers and accompanying leaves) were taken from each replicate and sampling date.

Plant material washings were obtained as described above by homogenizing blossoms and leaves with 30 ml of 50 mM sterile PBS (pH 7.0) and 0.1% peptone at 130 rpm for 30 min on ice bath. Plant material washings from field experiments were 10-fold concentrated by centrifugation at 10,000 × *g* for 10 min of 20 ml of plant extract and resuspended in 2 ml of sterile PBS and 0.1% peptone. The population levels of PM411 on blossoms and leaves were determined using qPCR, v-qPCR, and plate counting.

For qPCR, DNA was isolated from 1 ml of each plant material washing, as explained above. DNA was evaluated in triplicate using the TaqMan-based qPCR assay B (PM411-for, PM411-pr, and PM411B-rev, 188 bp). The quantification was performed by means of a standard curve of the corresponding plant material washing (apple or pear blossoms or strawberry, kiwifruit, or pear leaves) specifically developed (linear range of 1 × 10^3^ to 1 × 10^7^ CFU · ml^−1^, *R*^2^ = 0.99, *E* >80%), and used in each plate run. The amount of total cells by qPCR was obtained by interpolating the *C_T_* values from the unknown samples against the corresponding developed standard curve and expressed as log_10_ CFU per blossom or leaf.

For v-qPCR, previously to DNA isolation, 1 ml of sample was treated with PEMAX at 50 μM, according to the procedure described above. DNA extraction, qPCR assay, and quantification, using the corresponding standard curve with PEMAX treatment (linear range of 1 × 10^3^ to 1 × 10^7^ CFU · ml^−1^, *R*^2^ = 0.99, and *E* >80%), were carried out as described above.

For plate counting, plant material washings were serially diluted, and appropriate dilutions were seeded using a spiral plater (Eddy Jet; IUL Instruments, Barcelona, Spain) onto MRS agar plates supplemented with 50 μg · ml^−1^ rifampin (Sigma) to counterselect PM411 and 10 μg · ml^−1^ econazole nitrate salt (Sigma) to avoid fungal growth. Plates were incubated at 30°C for 48 h, and colonies were counted using an automatic counter system (Countermat Flash; IUL Instruments). The culturable population level of PM411 was expressed as the log_10_ CFU per blossom or leaf.

### Statistical analysis.

To test the significance of the effect of PEMAX concentration and qPCR design in the suppression of DNA amplification (signal reduction) on dead and viable cells of PM411, a two-way analysis of variance (ANOVA) was performed. To test the effect of the quantification method to estimate the PM411 population on plant surfaces for each sampling date, ANOVA was performed. Means of the Δ*C_T_* (signal reduction) or CFU · blossom^−1^ or CFU · leaf^−1^ (population) were separated according to the Tukey's test at a *P* value of ≤0.05. The statistical analyses were performed using GLM procedure of the PC-SAS (version 9.1; SAS Institute, Inc., Cary, NC).

### Accession number(s).

The putative phage sequence of L. plantarum strain PM411 has been deposited in the GenBank database under the accession number MG788324.

## Supplementary Material

Supplemental material
