# Supplementary material for: Monitoring Viable Cells of the Biological Control Agent Lactobacillus plantarum PM411 in Aerial Plant Surfaces by Means of a Strain-Specific Viability Quantitative PCR Method
Source: Appl Environ Microbiol. 2018 May 1;84(10):e00107-18. doi: 10.1128/AEM.00107-18 (PMC5930365; doi:10.1128/AEM.00107-18)
Supplement: Supplemental material [file supp_84_10_e00107-18__index.html]

Supplemental material 

# Monitoring Viable Cells of the Biological Control Agent Lactobacillus plantarum PM411 in Aerial Plant Surfaces by Means of a Strain-Specific Viability Quantitative PCR Method

## Supplemental material

- Supplemental file 1 -

  Characteristics of the putative 69.6-Kb prophage in the *Lactobacillus plantarum* PM411 genome according to the PHAST bioinformatic tool (Table S1).

  PDF, 11K
