# Supplementary material for: Monitoring Viable Cells of the Biological Control Agent Lactobacillus plantarum PM411 in Aerial Plant Surfaces by Means of a Strain-Specific Viability Quantitative PCR Method
Source: Appl Environ Microbiol. 2018 May 1;84(10):e00107-18. doi: 10.1128/AEM.00107-18 (PMC5930365; doi:10.1128/AEM.00107-18)
Supplement: Supplemental material [file AEM.00107-18_zam010188502s1.pdf]

Table S1. Characteristics of the putative prophage of 69.6 Kb (GenBank accession number MG788324) in *Lactobacillus plantarum* PM411 genome according to PHAST bioinformatic tool.

#	CDS POSITION	BLAST HIT [1]	E-VALUE
1	1..12	attL ATAAAATAAAAA	0
2	complement(9359..10516)	PHAGE_Lactob_Lj965_NC_005355: putative integrase; PP_00012; phage(gi41179218)	2.00E-46
3	complement(10686..11354)	PHAGE_Sulfit_NYA_2014a_NC_027299: T5ord172-domain containing protein; PP_00013; phage(gi848469691)	4.00E-05
4	complement(11466..11897)	nucleotide-binding protein, UspA family [Lactobacillus plantarum JDM1]. gi|254555641|ref|YP_003062058.1|; PP_00014	2.00E-75
5	complement(11997..12254)	hypothetical; PP_00015	0
6	complement(12378..12554)	hypothetical protein LPST_C1709 [Lactobacillus plantarum subsp. plantarum ST-III]. gi|308180891|ref|YP_003925019.1|; PP_00016	3.00E-21
7	complement(12581..12670)	tRNA	0
8	complement(12724..13284)	hypothetical; PP_00017	0
9	complement(13376..13807)	PHAGE_Lactob_phig1e_NC_004305: hypothetical protein; PP_00018; phage(gi23455774)	2.00E-31
10	complement(13817..14191)	PHAGE_Lactob_phig1e_NC_004305: repressor; PP_00019; phage(gi23455773)	1.00E-22
11	14513..14722	PHAGE_Strept_phiARI0746_NC_031907: hypothetical protein; PP_00020; phage(gi100038)	1.00E-06
12	14726..14929	hypothetical protein JDM1_0477 [Lactobacillus plantarum JDM1]. gi|254555646|ref|YP_003062063.1|; PP_00021	1.00E-25
13	14929..15201	hypothetical protein JDM1_0478 [Lactobacillus plantarum JDM1]. gi|254555647|ref|YP_003062064.1|; PP_00022	1.00E-43
14	15339..15788	PHAGE_Lister_B025_NC_009812: gp52; PP_00023; phage(gi157325269)	3.00E-39
15	15775..15951	Hypothetical protein zj316_1378 [Lactobacillus plantarum ZJ316]. gi|448820901|ref|YP_007414063.1|; PP_00024	3.00E-21
16	15944..16129	hypothetical protein JDM1_0480 [Lactobacillus plantarum JDM1]. gi|254555649|ref|YP_003062066.1|; PP_00025	4.00E-28
17	16101..16271	Hypothetical protein zj316_1380 [Lactobacillus plantarum ZJ316]. gi|448820903|ref|YP_007414065.1|; PP_00026	4.00E-18
18	16274..16591	hypothetical; PP_00027	0
19	16579..16698	Hypothetical protein zj316_1381 [Lactobacillus plantarum ZJ316]. gi|448820904|ref|YP_007414066.1|; PP_00028	7.00E-09
20	16695..17174	PHAGE_Lactob_LfeSau_NC_029068: hypothetical protein; PP_00029; phage(gi985757759)	7.00E-33
21	17245..18585	PHAGE_Lactob_PLE2_NC_031036: hypothetical protein; PP_00030; phage(gi100040)	7.00E-153
22	18657..19298	PHAGE_Lactob_PLE2_NC_031036: hypothetical protein; PP_00031; phage(gi100041)	1.00E-63
23	19301..19924	PHAGE_Lactob_PLE2_NC_031036: hypothetical protein; PP_00032; phage(gi100042)	9.00E-30
24	19995..20789	PHAGE_Lactob_PLE2_NC_031036: hypothetical protein; PP_00033; phage(gi100043)	1.00E-74
25	20786..22060	PHAGE_Lactob_PLE2_NC_031036: hypothetical protein; PP_00034; phage(gi100044)	9.00E-123
26	22318..22659	phage protein [Lactobacillus plantarum JDM1]. gi|254555659|ref|YP_003062076.1|; PP_00035	3.00E-57
27	22640..22837	hypothetical; PP_00036	0
28	22830..23141	hypothetical protein Lp16_1925 [Lactobacillus plantarum 16]. gi|513841770|ref|YP_008121742.1|; PP_00037	8.00E-53
29	23145..23303	PHAGE_Lactob_Sha1_NC_019489: hypothetical protein; PP_00038; phage(gi418489791)	1.00E-09
30	23306..23431	hypothetical protein JDM1_0491 [Lactobacillus plantarum JDM1]. gi|254555660|ref|YP_003062077.1|; PP_00039	7.00E-14
31	23664..23954	PHAGE_Weisse_WCP30_NC_031101: XRE family transcriptional regulator; PP_00040; phage(gi100024)	3.00E-08
32	23951..24409	PHAGE_Lactob_Sha1_NC_019489: hypothetical protein; PP_00041; phage(gi418489796)	5.00E-22
33	24649..24960	hypothetical protein JDM1_0494 [Lactobacillus plantarum JDM1]. gi|254555663|ref|YP_003062080.1|; PP_00042	6.00E-52
34	24972..25385	Prophage protein [Lactobacillus plantarum ZJ316]. gi|448820916|ref|YP_007414078.1|; PP_00043	3.00E-71
35	25501..26490	hypothetical; PP_00044	0
36	26571..27293	hypothetical; PP_00045	0
37	27508..27918	PHAGE_Entero_phiEf11_NC_013696: SbcC domain protein; PP_00046; phage(gi282598758)	3.00E-12
38	27985..28200	PHAGE_Lactob_phig1e_NC_004305: hypothetical protein; PP_00047; phage(gi254854752)	8.00E-08
39	28184..28522	PHAGE_Lister_B025_NC_009812: gp65; PP_00048; phage(gi157325282)	7.00E-25
40	28522..28764	Prophage protein [Lactobacillus plantarum ZJ316]. gi|448820920|ref|YP_007414082.1|; PP_00049	3.00E-32
41	28782..29024	Prophage protein [Lactobacillus plantarum ZJ316]. gi|448820921|ref|YP_007414083.1|; PP_00050	6.00E-37
42	29132..29419	PHAGE_Bacill_vB_BhaS_171_NC_030904: putative Cro/Cl-type repressor; PP_00051; phage(gi100005)	1.00E-12
43	29416..31098	PHAGE_Bacill_vB_BhaS_171_NC_030904: XRE family transcriptional regulator; PP_00052; phage(gi100006)	3.00E-141
44	31117..32259	PHAGE_Staphy_P954_NC_013195: HK97 family phage portal protein; PP_00053; phage(gi257136404)	1.00E-93
45	32246..32998	PHAGE_Lactob_PLE2_NC_031036: hypothetical protein; PP_00054; phage(gi100004)	4.00E-45

46	33019..34197	PHAGE_Lister_LP_101_NC_024387: major capsid protein; PP_00055; phage(gi658607815)	5.00E-101
47	34336..34641	PHAGE_Bacill_vB_BhaS_171_NC_030904: endonuclease; PP_00056; phage(gi100011)	5.00E-10
48	34622..35011	PHAGE_Lister_B025_NC_009812: gp8; PP_00057; phage(gi157325226)	2.00E-20
49	35008..35415	PHAGE_Lister_LP_101_NC_024387: hypothetical protein; PP_00058; phage(gi658607819)	1.00E-14
50	35412..35834	PHAGE_Lister_LP_101_NC_024387: hypothetical protein; PP_00059; phage(gi658607820)	9.00E-15
51	35849..36454	PHAGE_Staphy_6ec_NC_024355: major tail protein; PP_00060; phage(gi658310345)	1.00E-21
52	36429..36701	PHAGE_Salmon_BP63_NC_031250: terminase small subunit; PP_00061; phage(gi100030)	6.00E-10
53	36773..37087	hypothetical protein JDM1_0510 [Lactobacillus plantarum JDM1]. gi|254555679|ref|YP_003062096.1|; PP_00062	1.00E-47
54	37111..37332	Prophage protein, tail tape measure protein [Lactobacillus plantarum ZJ316]. gi|448820933|ref|YP_007414095.1|; PP_00063	3.00E-32
55	37351..41895	PHAGE_Lactob_LF1_NC_019486: phage tail tape measure protein; PP_00064; phage(gi418489397)	0
56	41899..42720	PHAGE_Entero_phiFL1A_NC_013646: tail protein; PP_00065; phage(gi281416378)	3.00E-31
57	42740..46666	PHAGE_Lactob_ATCC8014_NC_019916: prophage tail super family protein; PP_00066; phage(gi431809813)	8.00E-75
58	46689..47174	PHAGE_Brocho_BL3_NC_015254: gp20; PP_00067; phage(gi327409412)	2.00E-09
59	47176..47610	hypothetical protein JDM1_0516 [Lactobacillus plantarum JDM1]. gi|254555685|ref|YP_003062102.1|; PP_00068	1.00E-74
60	47627..48010	PHAGE_Lactob_LfeSau_NC_029068: hypothetical protein; PP_00069; phage(gi985757745)	8.00E-32
61	48013..48207	PHAGE_Lactob_phijl1_NC_006936: hypothetical protein; PP_00070; phage(gi62327114)	3.00E-05
62	48207..48491	PHAGE_Lactob_phig1e_NC_004305: holin; PP_00071; phage(gi23455817)	1.00E-06
63	48491..49423	PHAGE_Entero_EF62phi_NC_017732: endolysin type Endo-N-acetylmuramidase; PP_00072; phage(gi384519788)	2.00E-40
64	49669..50103	PHAGE_Lactob_Lv_1_NC_011801: portal protein; PP_00073; phage(gi219563200)	2.00E-21
65	50066..51916	PHAGE_Lactob_Lv_1_NC_011801: protease-scaffold-major head protein; PP_00074; phage(gi219563201)	2.00E-65
66	52061..52378	hypothetical protein JDM1_0989 [Lactobacillus plantarum JDM1]. gi|254556156|ref|YP_003062573.1|; PP_00075	9.00E-33
67	52365..52712	PHAGE_Strept_Sfi19_NC_000871: putative head-tail joining protein; PP_00076; phage(gi9632902)	2.00E-11
68	52715..53119	PHAGE_Strept_DT1_NC_002072: putative tail component protein; PP_00077; phage(gi9632427)	3.00E-29
69	53119..53499	PHAGE_Strept_Sfi21_NC_000872: putative tail component protein; PP_00078; phage(gi9632947)	8.00E-16
70	53516..54169	phage major tail protein [Lactobacillus plantarum subsp. plantarum ST-III]. gi|308180857|ref|YP_003924985.1|; PP_00079	6.00E-113
71	54245..54619	hypothetical protein PEPE_0995 [Pediococcus pentosaceus ATCC 25745]. gi|116492758|ref|YP_804493.1|; PP_00080	8.00E-61
72	54892..59643	PHAGE_Strept_DT1_NC_002072: putative tail component protein; PP_00081; phage(gi29165636)	6.00E-176
73	59720..61495	PHAGE_Lactob_Ldl1_NC_026609: distal tail protein; PP_00082; phage(gi764162085)	1.00E-48
74	61559..63970	PHAGE_Lactob_Ldl1_NC_026609: baseplate protein tal-like protein; PP_00083; phage(gi764162086)	4.00E-129
75	63988..66228	PHAGE_Lactob_LP65_NC_006565: tail fiber; PP_00084; phage(gi56693145)	2.00E-139
76	66221..66463	PHAGE_Lactob_Sha1_NC_019489: hypothetical protein; PP_00085; phage(gi418489818)	3.00E-33
77	66467..66628	PHAGE_Lactob_Sha1_NC_019489: hypothetical protein; PP_00086; phage(gi418489819)	3.00E-22
78	66612..67118	PHAGE_Lactob_Sha1_NC_019489: prophage Lp2 protein 53-like protein; PP_00087; phage(gi418489820)	2.00E-68
79	67124..67594	PHAGE_Lactob_iLp1308_NC_028911: tail fiber protein; PP_00088; phage(gi971754939)	1.00E-13
80	67606..68634	PHAGE_Lactob_PLE3_NC_031125: hypothetical protein; PP_00089; phage(gi100023)	1.00E-79
81	68634..68903	PHAGE_Lactob_Sha1_NC_019489: phage-related holin; PP_00090; phage(gi418489822)	6.00E-32
82	68915..69277	PHAGE_Lactob_LP65_NC_006565: hypothetical protein; PP_00091; phage(gi56693137)	4.00E-23
83	69638..69649	attR ATAAAATAAAAA	0

[1]: Hits against Virus and prophage DB (marked in grey) and hits against Bacterial DB or GenBank file (not marked)
